# Mapping potential community resources and care pathways for earlier identification and recovery-oriented intervention for people with psychosis in Addis Ababa, Ethiopia

**DOI:** 10.1371/journal.pone.0297891

**Published:** 2026-02-18

**Authors:** Mekonnen Tsehay, Teshome Shibre Kelkile, Wubalem Fekadu, Alex Cohen, Eleni Misganaw, Charlotte Hanlon

**Affiliations:** 1 Department of Psychiatry and WHO Collaborating Centre for Mental Health Research and Capacity-Building, School of Medicine, College of Health Sciences, Addis Ababa University, Addis Ababa, Ethiopia; 2 Horizon Network Zone 3, Toronto, NB, Canada; 3 Department of Psychiatry, Dalhousie University, Halifax, NS, Canada; 4 Department of Population Health, London School of Hygiene & Tropical Medicine, London, United Kingdom; 5 Mental Health Service Users Association, Addis Ababa Ethiopia; 6 Division of Psychiatry, Centre for Clinical Brain Sciences, University of Edinburgh, Edinburgh, Scotland, United Kingdom; 7 Centre for Innovative Drug Development and Therapeutic Trials for Africa, College of Health Sciences, Addis Ababa University, Addis Ababa, Ethiopia; National Institute of Mental Health and Neurosciences: National Institute of Mental Health and Neuro Sciences, INDIA

## Abstract

**Background:**

There is a pressing need to reduce the long duration of untreated illness and improve care and outcomes for people with psychosis in Ethiopia. This study aimed to map community resources that have the potential to be leveraged to achieve earlier and more recovery-oriented interventions for people with psychosis in Addis Ababa, Ethiopia.

**Method:**

A strength-based resource mapping exercise was undertaken in two sub-cities, covering an estimated population of half a million people. We identified the types of resources to be mapped, based on their importance for multi-sectoral care in mental health: healthcare facilities, religious organisations, traditional and faith healers, non-governmental organisations (NGOs), and social/community organisations. The lead investigator traversed the study sites to gather information on community resources, recorded the Global Positioning System (GPS) coordinates of the resources, and consulted with key informants. The information obtained was complemented by a participatory Theory of Change workshop attended by 30 stakeholders.

**Results:**

We identified 124 health facilities, of which only 16 health centres and nine hospitals currently provide mental health services. We identified three registered traditional healers, 38 religious organisations, 104 non-governmental organisations, and other charitable/community-based organisations. In addition, three health facilities, six holy water religious healing sites, and four traditional healers were identified as out-of-site resources that were popular and frequently visited by people living in the sub-cities. The two sub-cities also had six feeding centres each providing meals for 1000 people in need. There were extensive networks of social organisations and community-based associations. Existing care pathways are complex but commonly include traditional and religious healing sites as places of first contact.

**Conclusions:**

We identified important available resources that provide a wealth of opportunities for improving the early identification and outcomes of people with psychosis.

## Introduction

In low- and middle-income countries (LMICs), the majority of people with psychosis either do not access any evidence-based care or experience substantial delays in receiving treatment [1]. An estimated 70%−90% of people with psychosis in need of biomedical mental health care in LMICs do not receive it [2,3]. There are no estimates of the prevalence of psychosis in Addis Ababa. However, in rigorous community-based studies conducted in Ethiopia, the prevalence of severe MHCs, specifically for schizophrenia, was (lifetime) 0.5% [4], and for bipolar disorder was (lifetime) 0.5% [5], and the median duration of untreated illness in people with psychosis was more than five to seven years [6,7]. Even when care is accessed, it is often narrowly biomedical and fails to address prominent social and economic needs [8,9].

The consequences of absent, delayed, or inadequate care for people with psychosis are substantial in terms of impairing a person’s capacity to function in social, familial, and economic domains. Furthermore, stigma, prejudice, and violations of human rights are commonly experienced by persons with psychosis, exacerbated by a lack of effective treatment [8,10–12].

Although there are multiple factors contributing to treatment delays and the long duration of untreated psychosis, the most frequently cited relate to the demand side (e.g., financial constraints, stigma, differing explanatory models of illness) and supply-side factors (e.g., lack of available, accessible and adequate mental health services) [13,14]. Consequently, help-seeking pathways are often convoluted and may involve multiple contacts with traditional and/or faith healers or non-specialist healthcare providers before accessing mental health services [15,16]. Even when mental health services are accessed, engagement is often intermittent and poorly coordinated with pluralistic approaches to care [17,18]. There is evidence that earlier and better interventions for people with psychosis lead to better health and social outcomes, as well as lower costs [19–21].

Given the limited accessibility, workforce shortages, and stigma associated with facility-based mental health services, leveraging community resources, such as social networks, religious institutions, and local health initiatives, offers a culturally grounded and potentially more sustainable approach to supporting individuals with mental illness [22].

The World Health Organization (WHO) has published guidance on person-centered and rights-based approaches to community mental health care, recommending the inclusion of communities to support pluralism of care and the provision of psychosocial interventions to augment biomedical care [23]. This community-focused approach first requires an understanding of the potential contributions of both formal biomedical care and informal community-based traditional and religious healers, as well as organisations supporting social and economic well-being, to build on existing resources when designing interventions. In a previous study from rural Ethiopia, strengths-based mapping in a rural district identified rich community resources [24] that formed an input into the design of models of care for people with psychosis [25,26]. This included the PRIME project (Programme for Improving Mental Health CarE) in Ethiopia, where participatory methods were used to develop a district mental health care plan to expand access to mental health care within primary healthcare services [27]. Evidence from urban African settings illustrates the promise of community-based mental health models. In Kenya’s urban congregational settings, community-embedded interventions delivered through religious groups have effectively reached underserved families [28].

Drawing on the identified existing community resources, inter-linked interventions at the level of community, facility, and health system were developed and implemented, leading to improved functioning, better socio-economic status, and reduced experiences of discrimination and abuse [6,29]. Mapped community resources were also integral to the interventions and approaches of the Ethiopia RISE study (Rehabilitation Intervention for people with Schizophrenia in Ethiopia) [30], which showed that community-based rehabilitation led to improved functioning in people with psychosis who had not responded to primary healthcare-based mental health care [31]. Recovery-oriented interventions emphasise hope, empowerment, and the active involvement of individuals in their own healing process, shifting the focus from symptom reduction to meaningful life outcomes [32]. This approach is particularly relevant in low-resource settings, where formal services are limited and leveraging existing community resources and mapping these strength-based resources is essential to inform more contextually grounded, sustainable, and person-centred mental health care systems [33,34].

Building on this asset-oriented approach, health‑assets theory conceptualises community‑level resources such as social capital, cultural capital, and resilience as foundational to mental well-being [35]. For instance, A multi-country qualitative study led in Ghana, India, Palestine, and South Africa showed how informal mutual support networks, peer-based encouragement, and faith/traditional healer involvement play a vital role in strengthening engagement and fostering belonging [36]. In South Africa, systematic reviews of collaborative care models have highlighted the inclusion of lay screening and partnership with traditional healers as culturally congruent and effective community‑based strategies [37].

Evaluation of efforts to increase access to mental health care in Ethiopia has focused on rural settings. There may be distinct opportunities and challenges in the capital city of Addis Ababa; for example, in terms of greater availability and accessibility of mental health services, concentration of NGOs, diverse community and faith-based organisations, and greater proximity to formal health facilities. but also the potential for fragmentation of care, more transient populations, less social capital, substance use, and a visible population of people who are homeless and have apparent psychosis [38]. There is limited existing research or data on the availability of community-based resources and how they might be harnessed to support recovery of people with psychosis in Addis Ababa. Localised knowledge is essential for intervention planning that builds on existing strengths. Therefore, this study aimed to map potential community resources and care pathways for earlier identification and recovery-oriented intervention for people with psychosis in Addis Ababa, Ethiopia. Specifically, the study sought to: (1) To identify and map the range of formal, religious, community-based, and traditional resources that are potentially involved in the early identification, referral, or support of people with psychosis in Addis Ababa, and (2) To examine existing care pathways, including help-seeking patterns, referral flows, and linkages, between community resources and formal mental health services. More broadly, the findings underscore the importance of considering local contexts and community assets when developing interventions for psychosis in global mental health.

## Methods

### Study design

We conducted a strength-based resource mapping exercise from 13^th^ September to 10^th^ October 2022. This approach includes exploring, describing, and mapping community resources before employing these resources to develop solutions to a specific problem [39]. Underpinning this method is a strengths-based orientation that seeks to build on the strengths of individuals, families, and communities [40]. Several other frameworks exist for resource or asset mapping, including the Asset-Based Community Development (ABCD) model [41], Morgan and Ziglio’s Asset Model for Health [42], WHO’s SARA approach for health service availability [43], GIS-based spatial mapping frameworks [44], and participatory methods such as PRA/PLA [45]. We selected the six-step framework by Turin et al. (2019) because it offers a systematic and practical structure aligned with our study aims and urban health system context.

What makes the strengths-based approach different is that it focuses on capabilities and existing community solutions that are grounded in values and collective action, while more deficit-focused approaches (e.g., Needs-Based Assessment) addresses needs and deficits, utilizing services to address problems [46]. Similar to Asset-Based Community Development (ABCD) [41] and Participatory Rural Appraisal (PRA) [45], strengths-based resource mapping involves the community and stakeholders in the process of resource identification and assessment, which can help to increase buy-in and ensure more sustainable and acceptable interventions [47,48]. Unlike PRA, community members were not involved as data collectors or the analysis, but they did review the findings in advisory board meetings and theory of change workshops. Distinct from ABCD, findings from formal research activities in the larger SCOPE study [49] will be an additional input into participatory intervention planning.

The six-step framework by Turin et al. (2019) offers a systematic and adaptable structure suitable for our study’s aim of identifying and characterising diverse formal, religious, and community-based resources within an urban setting.

The study was nested within the formative phase of a larger project, SCOPE (Studying the Contexts of Recent Onset Psychoses in Ethiopia) [49]. SCOPE aims to produce contextual evidence about psychosis and uses participatory methods to design and evaluate innovations to achieve earlier detection and care to improve the lives of people living with psychosis in rural and urban settings in Ethiopia.

### Setting

The urban sites included in SCOPE are Lideta and Kirkos sub-cities of Addis Ababa, the capital city of Ethiopia. According to the Central Statistical Agency of Ethiopia (CSA), in 2022 [50], the projected population of the sub-cities is 595, 973 (134,372 males, and 149836 females in Lideta and 144,461 males and 167,304 females in Kirkos). The total area of the sub-cities is 52,284.1 square kilometres. Both sub-cities are among the most densely populated and socioeconomically diverse areas, characterised by a mix of residential and commercial zones, alongside significant levels of economic vulnerability.

The two sub-cities were selected purposively for the SCOPE project because both Lideta and Kirkos are inner-city areas with diverse populations and significant unmet mental health needs. Our team has existing collaborations with the Lideta sub-city (e.g., through the SafeHome project [38]) and earlier primary care mental health initiatives. Kirkos was selected for its higher levels of socioeconomic deprivation, with numerous nightclubs and prominent substance use; factors that are known to increase the risk of psychosis and contribute to delayed or fragmented help-seeking. Together, these sub-cities offer a contextually rich environment for mapping both formal and informal resources, potentially to the early identification and support of individuals experiencing psychosis.

### Procedures

Resource mapping processes are flexible. Our approach aligned with the six steps of Turin TC, et al. (2019), referred to as the “Bird’s eye view of the asset map creation process” [51]: 1) Determine the purpose of mapping: we first determined the research questions and objectives, drawing on previous work in Ethiopia and in consultation with stakeholders. 2) Define community boundaries: we decided to map the resources of the two sub-cities of Addis Ababa, Kirkos, and Lideta (SCOPE project study area). 3) Identify and engage stakeholders: we engaged with members of the community advisory board of the SCOPE project as gatekeepers and as additional key informants. The advisory board members were selected based on their public positions of influence/knowledge in relation to mental health (e.g., health administrators, government officials in the social affairs office, police, health workers, NGO workers, religious leaders, lived experience experts) 4) Determine what resources to include: Building on a meeting with the Community Advisory Board, the research team held a series of internal discussions to identify which types of resources were most relevant for mental health support in the study area. We prioritised mapping key resources such as healthcare facilities, religious organisations, traditional and faith healers, non-governmental organisations (NGOs), and social or community-based groups. Additional resources were also included when identified through the lead investigator’s community walks and observations. Key informant interviews further helped pinpoint the places people commonly seek help or healing when experiencing mental health problems, ensuring the inventory captured both formal and informal support pathways. We did not apply strict inclusion or exclusion criteria for selecting resources. Any site or service direct or indirect relevance to people with severe mental illness, and demonstrated use or importance within the community’s help-seeking pathways. Accordingly:

1. All selected resources within the catchment area: health facilities, religious institutions, traditional and faith-healing sites, NGOs, and community organisations, were included if they were known to provide any form of support, clinical, spiritual, social, or cultural: Adstionaly, resources with the potential to be relevant were considered even if they were not currently providing support to individuals experiencing mental health problems.
2. Highly frequented or widely recognised resources outside the catchment area (e.g., Entoto St. Mary holy water site; Emanuel Mental Health Specialised Hospital) were also included when key informants and community advisory board members consistently identified them as important destinations for psychosis-related care or healing for people residing in Addis Ababa.

The identification of these resources was informed by community walks conducted by the lead investigator, key informant interviews, and consultation with the Community Advisory Board, which collectively ensured that the selected sites were actually used or perceived as relevant by people with psychosis and their families.

5) Creating an Inventory of Resources: Creating a comprehensive inventory of resources was an interactive and iterative process that combined formal record review, institutional engagement, and community-based exploration.

A First, we visited sub-city administrative offices to obtain official permission letters, which facilitated access to relevant sector offices and organisations. We then engaged with the health offices in each sub-city to gather foundational information on the availability and distribution of health facilities, including health centres, private clinics, and hospitals.
B In parallel, we identified sub-city-level NGOs and feeding centres to understand their presence and potential contributions to mental health and psychosocial support.
C At the district (woreda) level, we worked closely with health offices to document the number of registered traditional healers and NGOs and to explore the specific roles they play in mental health care initiatives, particularly in community-based support and early referral.
D Recognising that community assets extend beyond health-focused organisations, we expanded our mapping to include the social affairs departments in each district. These offices helped us identify key community associations, such as youth groups, women’s groups, and disability associations, that often serve as important points of engagement, support, and information-sharing for local residents.
E To complement these formal data sources, the lead investigator conducted community walks across the study sites. These walks allowed for direct observation, the identification of additional community resources, and we noted locations.
F Informal consultations with community members and key informants were an integral part of this process, helping uncover where people commonly seek help for mental health concerns, the perceived roles of various organisations, and the informal networks that operate beyond official registries. A snowballing approach naturally emerged, as key informants highlighted additional resources, individuals, and organisations that warranted further exploration. We asked: where do people go for healing if they faced a mental health problem? While administrative records provided a valuable starting point, they did not capture the full breadth of relevant resources, particularly informal support structures and religious organisations, and community/social organisations, Edir, Tsiwa Mahber and Equb, which became visible only through this multi-step, iterative mapping process.

After compiling information from these multiple sources, we organised all identified resources into structured tables, allowing for clear comparison across sub-cities and districts. These tables included details such as the type of resource, services offered, geographic location, organisational affiliation, and any noted involvement in mental health care. Each entry was cross-checked for completeness and consistency.

**Fig 1:**
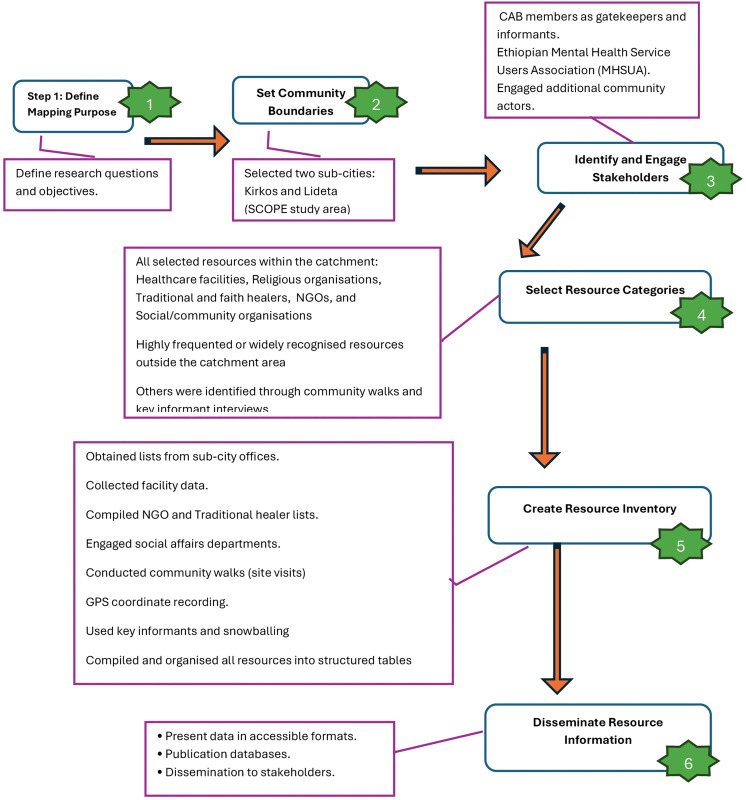
Resource mapping process (Adapted from Turin et al., 2019).

6) Organise resources for dissemination: Organise resources for dissemination through categorisation, visualisation, stakeholder-focused communication, and publication in an accessible database. Refer to Fig 1; it shows the simplified map creation process.

We then developed an initial list of resources based on: (1) community or organisation consultation; and (2) official lists of registered traditional healers and health facilities in each sub-city. Data were recorded about the total catchment population, mental health service provision (including the number of psychiatrists, mid-level mental health professionals, and psychologists), and number of general health workers who had been trained with the World Health Organization’s mental health Gap Action Programme (mhGAP) in line with the National Mental Health Strategy of Ethiopia [52]. We also recorded the presence of international or national charitable organisations and social organisations (*Edir* (burial groups, supporting saving) by searching through the districts of sub-cities.

The mapping information was complemented by a participatory Theory of Change (TOC) workshop attended by 30 stakeholders: The participants were people with lived experience of mental health conditions, family members, religious leaders, traditional healers, health administration officials, community-based health workers, police, social affairs officials, NGO representatives, and community leaders. Participants for the TOC workshops were mostly identified through the offices that they held, e.g., president of the traditional medicine organization, president of the mental health service user association. As there is no association to represent family members of people with psychosis, family members were identified by the psychiatric nurses of the health centres. Of ToC workshop attendees, 22 were men and 8 were women, reflecting the under-representation of women in administrative and leadership positions. The ToC workshop was conducted before initiating resource-mapping data collection as part of the broader SCOPE project. Its purpose was to articulate pathways to care, identify existing gaps, and define desired outcomes for early identification and treatment of psychosis. During these discussions, stakeholders identified a substantial treatment gap for people with severe mental illness and formulated a guiding question that directly informed our mapping approach:

“Where do people go when they experience a mental health challenge?”

This question shaped our community walks and key informant interviews and helped identify both formal and informal help-seeking sites. The TOC workshop aimed to obtain further information on pathways to care and existing opportunities for intervention to support people with psychosis to achieve valued outcomes. TOC workshop participants contributed to snowballing by identifying further key informants to approach and acted as gatekeepers to support our approaches to the districts.

The workshop was moderated by two facilitators. Meeting minutes from the ToC sessions were incorporated into our resource mapping data for analysis.

### Data management and analysis

Data were stored in Excel. We summarized the data descriptively (frequency and percentage).

### Ethical considerations

We received ethical approval from the Institutional Review Board of the College of Health Science, Addis Ababa University (084/11/PSY) and Kings College London (HR/DP-21/22–26183). We also obtained permission letters from each sub-city and verbal consent from each key informant.

## Results

Within each sub-city, we identified a wide array of community resources. These included health facilities, traditional healers, religious organisations, NGOs, and other charitable/community-based organisations (Table 1). Alongside this, we identified resources located geographically outside the sub-cities that were popular and frequently visited by people living in the sub-cities. The two sub-cities also had feeding centres, and social organisations: *Edir* (burial groups, where community members pay regular amounts of money to cover funeral costs and provide collective support for mourning ceremonies), *Equb* (small financial group where members pay in regular amounts of money and receive pay-outs on a rotating basis), *Tsiwa Mahiber* (mostly orthodox Christian religious groups where people come together to share coffee, drinks, and other refreshments. These gatherings are usually held to commemorate or celebrate miraculous events attributed to God, St. Mary, Angels, or other saints. They also serve as opportunities for community support, where assistance is provided to those in need), youth associations, women’s associations, elderly associations, and disability associations. Existing care pathways are complex but commonly include traditional and religious healing sites as places for both first contact and continuing contacts utilized alongside mental health services.

**Table 1:** Summary of Mental Health and Related Community Resources in Kirkos and Lideta Sub-Cities, Addis Ababa.

Resource Type	Facility/Provider	Location/Sub-city	Key Services Provided	Human Resources/ Support
Formal Health Facilities	124 health facilities,	Kirkos & Lideta	Only 16 health centres and nine hospitals currently provide mental health services	Psychiatric Nurses: 27 MSc Mental Health: 2 Psychiatrists: 34 Psychologists: 9 mhGAP-trained personnel vary (0–10 per site)
Religious Organizations	Ethiopian Orthodox Churches (11) Holy Water Sites (4) Muslim Mosques (9) Protestant Churches (13) Catholic Churches (2)	Kirkos & Lideta	Faith healing through prayer, holy water rituals, recitation of religious texts (Bible, Quran), and spiritual ceremonies	Conducted by priests, sheikhs, and lay religious leaders
Traditional Healers	Herbalists and diviners (3 identified)	Kirkos & Lideta	Herbal remedies, spiritual cleansing, bone setting, advice, and faith-based healing	Community-trusted individuals with no formal training
Feeding Centres	Supported by: Belayneh Kindie PLC, Tilahun Abateneh PLC, Medrok PLC, Hintsa College, Black Lion Hospital, Addis Ababa City Admin	Kirkos (in 4 woredas) Lideta (in 2 woredas)	Daily or weekly free meals are provided to vulnerable populations selected by district officials and churches	Target: 4000 beneficiaries/day (currently ~1000) Some volunteer-led (e.g., St. Lideta Church youth and donors)
Non-governmental organizations	104 non-governmental organisations	Kirkos & Lideta	None of them gives direct mental health services	Children, women, and disabled people other than mental illness are their target population
Community/ Social Organisations	Edir Associations (~51 per district; 100–620 households each)	Kirkos & Lideta	Funeral support; social, economic, and emotional support; member obligations	Strong social support network; potential community gatekeeper
Community/ Social Organisations	Tsiwa Mahber Groups (10–30 members each)	Kirkos & Lideta	Monthly prayer, singing, shared meals (Tsebel tsediq)	Psychosocial and spiritual support; community engagement

### Health sector

In Kirkos sub-city, there are eight public health centres, 59 private clinics, and five hospitals (three private, and two public). In Lideta sub-city, there are eight public health centres, 44 private clinics, and eight hospitals (five private, one public, the Federal police general hospital, and the Defense Forces general hospital). All public health centres and hospitals provide mental health services, except one health center in Kirkos.

### Primary mental health care

In the health centres, mental health care is provided in outpatient clinics by psychiatric nurses and masters-level mental health practitioners working alongside WHO mhGAP-trained general health care workers (nurses or health officers). Commonly the mhGAP-trained nurse or health officers were assigned to antiretroviral therapy (ART) and cancer clinic out-patient clinics. They consult mental health professionals when they see new people presenting with mental health conditions (Table 2). The patient flow is not more than 15 per month in each health centre for all mental health conditions combined, for a population served of around 35,000 people. In some health centres, mental health professionals participate in non-mental health managerial duties of the health centres rather than direct delivery of mental health care.

**Table 2:** Mental health service in health facilities of Kirkos and Lideta sub-cities.

Health facilities in sub-cities		District	Catchment populations	Psychiatric Nurse	MSc Mental health practitioners	Psychiatrist	Psychologist	mhGAP trained personnel
Health centres	HC-1	KSC-D 2	34400	1	0	0	0	2
	HC-2	KSC-D 3	18389	1	0	0	0	3
	HC-3	KSC-D 4	40,000	1	0	0	0	4
	HC-4	KSC-D 5	28999	1	0	0	0	1
	HC-5	KSC-D 6	16771					1
	HC-6	KSC-D 8	29430	0	0	0	0	0
	HC-7	KSC-D 9	28914	1	0	0	0	2
	HC-8	KSC-D 11	30108	1	0	0	0	0
	HC-9	LSC-D 1	25922	1	0	0	0	3
	HC-10	LSC-D 7	97,617	0	2	0	0	10
	HC-11	LSC-D 3	51,000	2	0	0	0	3
	HC-12	LSC-D 2	25684	1	0	0	0	5
	HC-13	LSC-D 9	31113	1	0	0	0	4
	HC-14	LSC-D 10	39763	2	0	0	0	3
	HC-15	LSC-D 8	48,000	1	0	0	0	3
	HC-16	LSC-D 4	25,817	1	0	0	0	0
**P**ublic hospital	GH-1	KSC-D 7	–	7	0	2	1	3
	GH-3	LSC-D 7	–	0	0	1	0	0
	GH-4	LSC-D 7	–	5	0	23	7	0
Private hospitals	PH-1	KSC-D 7	–	0	0	1	0	0
	PH-2	KSC-D 9	–	0	0	2	0	0
	PH-3	KSC-D 4	–	0	0	2	0	0
	PH-4	LSC-D 10	–	0	0	1	0	0
	PH-5	LSC-D 3	–	0	0	2	0	0
	PH-6	LSC-D 4	–	0	0	2	0	0
	PH-7	LSC-D 7	–	1	0	0	1	0

HC = health center, GH = Government/public hospital, PH = private hospital, mhGAP = mental health gap action programme, LSC-D = Lideta sub-city district, KSC-D = Kirkos Sub-city district.

### Hospital-level secondary and tertiary mental health services

Black Lion Comprehensive Specialized Hospital (Lideta sub-city) has an outpatient psychiatric clinic staffed with 23 psychiatrists, five psychiatric nurses, and seven clinical psychologists. Zewditu Memorial Referral Hospital (Kirkos sub-city) has an outpatient psychiatric unit, two crisis admission beds, and a four-bed unit for people with substance use disorders., staffed by two psychiatrists, seven psychiatric nurses, one psychologist, and three mhGAP trained professionals. Psychotherapy services are also provided in these hospitals.

The health facility numbers were allocated by the researchers.

In private hospitals, outpatient and in-patient mental health services are provided by part-time psychiatrists. The Federal Police Hospital and the Ethiopian Defense Force Hospitals have psychiatric services but currently, the service is only for the military.

Although outside the two sub-cities, referrals are made to Amanuel Mental Health specialized hospital, a dedicated public mental health hospital with 239 beds, 27 emergency beds, an in-patient substance use unit, and multiple outpatient clinics with 128, 000 people with MHCs seen per year. The two main private psychiatric clinics in Addis Ababa are also outside the sub-cities but accessed by inhabitants.

### Religious, traditional, and faith healer (TFH) sites

Within the two sub-cities, we identified three traditional healers (mostly herbalists but also practising spiritual or faith-based healing, diviner, giving counsel, and bone setting) and four holy water healing sites linked to the Ethiopian Orthodox Church. Holy water is typically the first port of call when a family member has a condition perceived to be triggered by a spiritual or supernatural problem, an attribution which is commonly linked to psychosis. People drink the holy water or are baptised. They may stay at the holy water site for extended periods where they are supported by family or overseen by a holy water attendant who is paid for the role (Table 3).

**Table 3:** Religious organisations and their mental health care provision.

Religious organisations	Religious healing practices
Ethiopian Orthodox Christian churches Eleven churches with/without holy water Four holy water healing sites	Pray and recite verses from the holy bible for the ill. Bless the water and administer it as holy water. Some have holy water sites visited by several people every day. E.g.: Tekle haymanot, Yohanis, Lideta and St. Mary, and St. Egziabher Ab holy water sites.
Nine Muslim mosques	The sheik recites verses from the holy Quran for the ill. Outside of our study area, at the Quran home, the holy Quran will be recited by a gifted person
Thirteen Protestant Christian churches	Advertise at least one day per week for faith healing programs.
Two Catholic churches	Priests, recite verses from the holy bible and give “kibakdus”, holy anointing for the ill.
Three Traditional healers	Traditional healers are often consulted by people with mental illness. They are mostly herbalists, but they practice also spiritual or faith-based healing, divining, advice on what to do, and bone setting)

There are also other popular holy water places outside the two sub-cities. People also use the most popular Muslim religious healing site outside of our study area, the Quran home. The holy Quran will be recited by a person recognized as having gifts of healing. There are 11 Ethiopian Orthodox Christian churches, 13 protestant churches, nine mosques, and two catholic churches in the sub-cities.

### Non-governmental and community-based organisations

There are more than 104 charitable organisations registered in the two sub-cities. Makedonia, Selihom, and Gergesenon charitable organisations provide social care and residential services to people with mental health conditions whose families cannot cope or who have no social support. They employ master’s and degree-level mental health care providers and also work in collaboration with local mental health services for case reviews by senior psychiatrists.

### Community/social organisations

Numerous community-based organisations exist. The first is *Edir*, mainly a funeral association that provides financial, practical, and emotional support when a family member dies. However, the role of *Edir* groups also includes the provision of support to members experiencing various social, economic, and health problems. Participation in an Edir requires regular payments, attendance at meetings and active participation in the social commitments supported by the *Edir*. There are an estimated 51 *Edir* associations in each district in the Lideta and Kirkos sub-cities. Each group has about 100–620 households as members. Individuals can be members of more than one *Edir* association.

*Tsiwa Mahber* is the second most common type of organization, specific to the Orthodox Christian church. Activities include group prayer, singing, and shared meals (called “Tsebel tsediq” in Amharic) taking place once per month in a member’s home or church compound. The name of a specific *Tsiwa Mahber* association is usually taken from a saint’s name. The number of members may range from 10 to 30.

In each of the two sub-cities, there are also many other associations, each with a specific focus: for young people, women, the elderly, and people with disabilities. Government representatives directly or indirectly coordinate these non-profit community-based associations. Associations for the elderly and those with disabilities aim to provide mutual support for members, provide them with more authority, and uphold their legal rights in society. Members will be the first to receive funding when it becomes available from relief organisations or the government. However, people with mental illness are not included within the definition of ‘disability’ used to determine membership which narrowly focuses on physical disability. Women’s associations work to advance women’s rights, increase their participation in family and community decision-making, and strengthen their economic position. The purpose of the youth associations is to promote participation in neighborhood events, access to skill development opportunities, and projects that generate income.

Social organizations and community-based associations play an important role in supporting individuals with mental health conditions and their families. The type and extent of support provided varies depending on the nature of the organization, and even within the same type, practices may differ significantly based on local norms, leadership dynamics, and member engagement.

For instance, Edir, traditional funeral associations, go beyond their primary function of supporting funerary rites by offering both emotional and financial assistance during health crises. When a member agrees to a request for help, contributions may be mobilized to support the family with expenses related to caregiving, such as accommodation at holy water sites, visits to individuals undergoing healing, or temporary household labor support in the caregiver’s absence.

Similarly, faith-based organizations, including Tsiwa Mahber and various religious youth groups, may advise families to seek care, accompany individuals to healing sites (and occasionally to health facilities), and promote adherence to healing or treatment plans through moral and emotional encouragement. Youth groups are particularly active in mobilizing community resources: Sunday school members in Orthodox churches, youth fellowships in Protestant congregations, and organized youth within mosques often collect donations from congregants and organize communal meals (such as breakfasts or lunches) to support individuals and families in need, including those experiencing homelessness or mental health challenges. These initiatives contribute to meeting basic needs, covering healing-related expenses, and offering psychosocial support through visits, donations, and acts of service.

### Feeding centres

The Addis Ababa city administration has established feeding centres throughout the city, to feed those who are unable to obtain a meal at least once per day. The program is coordinated by the city administration and funded by volunteers (Table 4).

**Table 4:** Feeding centres in the SCOPE study area (Kirkos and Lideta sub-cities of Addis Ababa).

Sub-city	Feeding centres	Fund/supported by	No of beneficiaries
Kirkos	District/woreda 4	1. Belayneh kindie PLC 2. Tilahun Abateneh building PLC. 3. Medrok PLC 4. Hintsa college 5. Black Lion Hospital 6. Addis Ababa city administration	Centres feed lunch every day for individuals selected by district officials on the bases of need. The plan is to feed up to 4000, but currently 1000 per day.
	District/woreda 5		
	District/woreda 9		
	District/woreda 10		
Lideta	District/woreda 9		
	District/woreda 10		
	St. Lideta church (district/woreda 10)	Sunday school students receive money from another volunteer	People around the church on Saturdays one lunch/week

We found that certain community-based organisations and associations are not commonly accessible for people with psychosis. For example, *Edir, Equb*, youth associations, women’s associations, elderly associations, and disability associations may either exclude explicitly (in the case of disability association) or through expectations of membership (e.g., when a person with psychosis cannot make payments or contribute their time for *Edir*) or through less overt discrimination related to stigma against mental illness. With respect to non-governmental and charitable organisations, target populations for the organization are usually circumscribed (e.g., for women and children) and often do not include people with psychosis. In feeding centres, people with psychosis were able to obtain meal cards from district officials, as long as they were not acutely unwell. Others could also request/apply for meal cards for them on their behalf.

### Theory of Change workshop

The initial sources of assistance for people with psychosis were identified as community resources, primarily religious healers, holy water sites, and a new outreach service from primary health care facilities (“family health teams”). To help with community-based case identification, access to care, and to support holistic recovery for a person with psychosis, collaboration and the establishment of referral systems were recommended.

## Discussion

In this study, we aimed to map available mental health care resources in two sub-cities of Addis Ababa to inform improved care planning for people with psychosis. We found extensive potential community resources in Addis Ababa, reflecting a rich sociocultural context even within this metropolis. We identified health facilities, registered traditional healers, religious organisations, and charitable/community-based organisations. We also found popular resources outside the two sub-cities. Feeding centres, social organisations youth associations, women’s associations, elderly associations, and disability associations were also considered important but not always available to people with psychosis and their families. Traditional and faith healing sites are commonly the places of first contact.

Similar to a resource mapping exercise conducted in a rural Ethiopian district, there are rich cultural and social resources that may contribute to the recovery of people with psychosis in Addis Ababa [24]. Our findings of many diverse community organisations indicated that ties to local community structures remain salient in this capital city, contrary to our expectations. Nonetheless, many community supports appear not to be fully accessible to people with psychosis. More in-depth research is required to understand the reasons for this and the potential role these organisations might play in recovery from mental illness. In rural areas, there is evidence that stigma and impoverishment fuel the exclusion of people with psychosis from community life [53,54]. Even disability organisations that would be expected to address the needs of people with psychosis define themselves narrowly as only catering to the needs of people with physical disabilities. A fruitful strategy may, therefore, focus on stigma reduction alongside advocacy directed at organizations and services that have the most potential to provide services for people with MHCs and their families. The recent Lancet Commission on ending mental health stigma and discrimination [55], identified social contact interventions involving people with lived experience of MHCs to be the most effective way to reduce stigma. In the SCOPE project, within which this study is nested, we are working with the Mental Health Service User Association of Ethiopia to develop such interventions to overcome exclusion.

Religious and traditional healing are integral aspects of care for many people with psychosis in Ethiopia. Our context mapping identified numerous and important healing sites within and around Addis Ababa where people with psychosis and their families seek help. Holy water sites linked to the Ethiopian Orthodox Church, Protestant churches, and Mosques all provided faith healing. Alongside religious approaches were a variety of traditional healers using herbs, medicines, and eclectic approaches to healing. Although mental health services in Addis Ababa are much more accessible than in rural areas, religious and traditional approaches are usually the first port of call. In an old study conducted at the main psychiatric hospital, Amanuel Specialized Mental Hospital, only 41% of service users came directly to the hospital, with the remaining trying alternatives first [15]. In Ethiopia, people with psychosis and their families commonly seek out biomedical mental healthcare services after investing a substantial amount of time and money in alternative places [15,16,56]. This concern was also raised by the stakeholders in our Theory of Change workshop. However, compared to rural areas, less is known about the experience and role of community-based traditional and faith-healing providers and how people with psychosis and their families navigate help-seeking journeys in Addis Ababa. A recent scoping review of Ethiopian studies found that many people with severe mental health conditions in Ethiopia initially seek care from Holy Water sites and other traditional or faith-based healers. These providers play a central role in help-seeking pathways, although the quality and safety of their practices vary, and some interventions may pose risks. Collaboration with biomedical services remains limited, typically occurring through occasional informal referrals and parallel care arrangements [57]. Ongoing efforts to establish collaborations between mental health services and Ethiopian Orthodox Christian Holy Water sites in Addis Ababa highlight the potential mutual benefits [58]. In this work, the need for mental health care outreach is apparent, as well as openness to two-way referral and exchange of expertise.

This study’s finding that only a small proportion of health facilities provide mental health services aligns with previous research from Ethiopia and other low- and middle-income countries (LMICs), which has consistently documented the limited integration of mental health into primary care [22,59]. These gaps in service availability persist despite national efforts to scale up mental health care, including the implementation of initiatives such as the WHO Mental Health Gap Action Programme (mhGAP).

Our mapping study also indicated that efforts to decentralize mental health care through integration in primary health care facilities have not yet achieved their potential in Addis Ababa.

At present the patient flow for mental health care in primary health care centres is very low relative to projected population needs, while the specialist mental health services are overwhelmed. There are many potential advantages to the integration of mental health in primary health care services: accessibility, efficiency, affordability, acceptability, and greater potential for care that attends to both physical and mental health needs. Previous studies from Ethiopia have shown high levels of premature mortality in people with psychosis which is mostly caused by poor physical health [60]. A study from Ethiopia examining the impact of the COVID-19 pandemic on mental health services concluded that more integrated, de-centralized mental health care would have made the mental health system more resilient and should be urgently prioritized [61].

In rural settings in Ethiopia, task-shared mental health care within primary health care for people with psychosis has been shown to be safe and effective [62] but sustainability is limited by system constraints, including staff turnover, medication supply interruptions, and the lack of ongoing support from secondary mental health care services [63]. In Addis Ababa, these system bottlenecks are less salient, but fragmentation of services is evident and currently undermines successful de-centralization. Nonetheless, the recent Ministry of Health initiative to develop “family health teams” could play a key role in improving care for people with psychosis. Family health teams provide community outreach from primary healthcare facilities, specifically focused on those who are vulnerable and would otherwise struggle to access care, including homeless populations. Given their prominent community footing, family health teams could contribute importantly to the early identification and ongoing engagement of people with psychosis in care. Previous research from South Africa indicates that outreach services can be an effective and efficient means to engage with people with mental illness and that this approach is less stigmatizing than clinic or outpatient-based care [64].

The private healthcare sector is important in Addis Ababa; 94% of health facilities are private [65]. Previous researchers have noted that private mental health care is a ‘hidden face’ of community mental health care [66], given scant attention within the field of global mental health. In the private sector, mental health services are provided by a range of practitioners, including general physicians, psychiatric nurses, master’s level mental health professionals and psychiatrists, usually working on a part-time or on-call basis. While they may assist in addressing the treatment gap, there may be variability in the quality of care and financial burdens that limit accessibility. The ongoing SCOPE study will endeavour to develop connections with private clinics, systematically investigate characteristics of people with psychosis accessing this resource and the care provided within these settings [49].

Although we followed robust methods to map the resources, there were some limitations to our study. We only included registered and well-known traditional and faith healers in the study area, but we assume that more traditional healers may exist. Given the lack of formal registration, it was also difficult to accurately enumerate all social organisations, such as *Tsiwa Mahber* and *Equb.* Other more informal initiatives, e.g., within neighbourhoods would also not have been captured. As Addis Ababa was undergoing reforms into new sub-cities and districts, it was difficult to achieve our original goal to create a map pinpointing the exact locations of resources. We also had limited capacity to systematically assess the extent of involvement of people with psychosis and their families in the diverse social structures within their communities. This study relied on consultations with key informants and snowballing approaches, which had the advantage of feasibility and timely completion of the mapping, While this approach allowed us to identify where key community resources are located and their potential roles in mental health care, it did not capture the detailed practices through which these actors identify psychosis, provide support, or coordinate with formal services. These process-level insights, such as early identification practices, outreach routines, and intersectoral interactions, require the kind of in-depth interviews and observational work that form the focus of the ethnographic study currently underway.

## Conclusions

We identified important community resources that may be used by people with psychosis. Since many people may have access to these resources, these approaches have potential to be scalable. However, outreach and engagement with religious and traditional healers, awareness-raising and stigma reduction efforts are needed to facilitate access for people with psychosis. Integrating community resources into mental health care, or vice versa, can be a comprehensive, cost-effective, and long-lasting strategy.

### Recommendation

Building on the findings of this resource-mapping study, there is a clear need for practical strategies to enhance service availability and coordination. Developing a collaborative care model represents a promising approach for scaling up access to mental health services. Complementary strategies could include targeted training and sensitisation of religious centres, NGOs, and private hospitals to facilitate effective two-way referral systems, as well as rationalising and optimising human resources across government health centres and hospitals. Together, these approaches can strengthen the integration and reach of mental health care in urban settings. A collaborative, co-production framework that honours the inputs of both formal and informal sectors could support more responsive and contextually relevant care.
